# Communication from the Secretary of the Stark Co. Med. Society

**Published:** 1854-02

**Authors:** 


TOULON, January 11th, 1854.

*To the Editors of the N. W. Medical and Surgical Journal.*

GENTLEMEN: I place the following matter before you, and shall be glad to see it inserted in your Journal with your opinion, and the opinion of your city society, at the latest in your Feb. No.

Truly yours,

THOS. HALL,

Sec. of the Stark County Med. Society.

Dr. A. is in attendance on Mrs. B., and on a certain day gives an unfavorable and fatal *prognosis*, with the remark that he has done all that he can do in the case—or something tantamount to it. The friends wish a Dr. C. to see her, and ask Dr. A. if he has any objection to it. Dr. A. consents to to it and immediately writes to Dr. C. an open letter for the friends to forward, in which he states that "the woman has had vaginitis, hysteritis, peritaritis, making a case of fearful and fatal import," and concluding with a request to Dr. C. to meet him (Dr. A.) at 12 o'clock the next day. The messenger that brought the letter of Dr. A. to Dr. C., brought an urgent request to Dr. C. from the husband of the patient and her father, that he should come down immediately, for in their opinion twenty-four hours was too long a time to delay a consultation in the case, it being extremely doubtful if the patient could survive that long. Dr. C. went down and prescribed for the case, and met Dr. A. at the appointed hour the next day. The Drs. were living about 9 miles each from the patient, and 6½ miles from each other—the call coming to Dr. C. at sunset or a little later.

The opinion of you and the profession, as far as it can be obtained, is requested on the following points:

Had the patient any right to send for a physician besides the one in attendance? Admitting the right, was Dr. C. in a different position to any other physician by a letter having been written to him by the attending physician? The friends being aware of the contents of the letter, did not their request for Dr. C's immediate attendance, and for him to disregard the letter all together, at once make the letter a nullity and virtually transfer the case from Dr. A. to Dr. C. Had Dr. C. a right to see the case, and seeing it, had he any right to prescribe?

[It is our opinion that Dr. C. should not have consented to see the patient before the hour appointed for the consultation, without giving or sending notice of his intentions to Dr. A., that the case was not "virtually transferred" from Dr. A. to Dr. C., and that Dr. C. had not the right to prescribe, excepting in case of great emergency.]—EDITORS.

